# Baseline and early response 2-[18F]FDG-PET/MRI for prediction of radiotherapy outcome in uterine cervical squamous cell carcinoma: a prospective single-center observational cohort study

**DOI:** 10.1186/s41824-024-00188-7

**Published:** 2024-03-01

**Authors:** Sara Strandberg, Joakim Jonsson, Maryam Zarei, Kristina Aglund, Lennart Blomqvist, Karin Söderkvist

**Affiliations:** 1 Department of Radiation Sciences, Diagnostic Radiology, Umea University, Umea, Sweden; 2 Department of Radiation Sciences, Radiation Physics, Umea University, Umea, Sweden; 3 Department of Radiation Sciences, Oncology, Umea University, Umea, Sweden; 4 Department of Molecular Medicine and Surgery, Karolinska Institutet, Solna, Sweden

**Keywords:** Uterine cervical neoplasms, Positron emission tomography, Magnetic resonance imaging, Outcome, Treatment

## Abstract

**Background:**

Should early response imaging predict tumor response to therapy, personalized treatment adaptations could be feasible to improve outcome or reduce the risk of adverse events. This prospective single-center observational study on 2-fluorine-18-fluoro-deoxy-glucose (2-[18F]FDG) positron-emission tomography/magnetic resonance imaging (PET/MRI) features aims to investigate the association between semantic 2-[18F]FDG-PET/MRI imaging parameters and outcome prediction in uterine cervical squamous cell carcinoma (CSCC) treated with radiotherapy.

**Results:**

Eleven study participants with previously untreated CSCC were examined with 2-[18F]FDG-PET/MRI at baseline and approximately one week after start of curative radiotherapy. All study participants had at least 24 months clinical follow-up. Two patients relapsed during the follow-up period. Reduced tumor size according to visual assessment was present in 9/11 participants (median change in sum of largest diameters (SLD) − 10.4%; range − 2.5 to − 24.6%). The size reduction was less pronounced in the relapse group compared to the no relapse group, with median change in SLD − 4.9%, versus − 10.4%. None of the reductions qualified as significantly reduced or increased in size according to RECIST 1.1., hence all participants were at this stage classified as non-responders/stable disease. Median baseline functional tumor volume (FTV) for the relapse group was 126 cm^3^, while for the no relapse group 9.3 cm^3^. Median delta FTV in the relapse group was 50.7 cm^3^, representing an actual increase in metabolically active volume, while median delta FTV in the no relapse group was − 2.0 cm^3^. Median delta apparent diffusion coefficient (ADC) was lower in the relapse group versus the no relapse group (− 3.5 mm^2^/s vs. 71 mm^2^/s).

**Conclusions:**

Early response assessment with 2-[18F]FDG-PET/MRI identified potentially predictive functional imaging biomarkers for prediction of radiotherapy outcome in CSCC, that could not be recognized with tumor measurements according to RECIST 1.1. These biomarkers (delta FTV and delta ADC) should be further evaluated.

*Trial registration* Clinical Trials, NCT02379039. Registered 4 March 2015—Retrospectively registered, https://classic.clinicaltrials.gov/ct2/show/study/NCT02379039.

## Background

Uterine cervical squamous cell carcinoma (CSCC) incidence has decreased in many European countries due to vaccines against human papilloma virus high risk variants 16 and 18, but still CSCC accounts for approximately 2.5% of all new cancer cases diagnosed in women, and for 2.4% of all cancer-related mortality in women (ECIS European Cancer Information System 2023).

According to the 2023 update of the European guidelines for the management of patients with cervical cancer, published by the European Society of Gynecological Oncology (ESGO), the European Society for Radiotherapy and Oncology (ESTRO) and the European Society of Pathology (ESP), diagnostic workup in CSCC includes pelvic examination and biopsy, and for > T1a tumor stages, also magnetic resonance imaging (MRI) (Cibula et al. 2023). In locally advanced cervical cancer (T1b3 and higher), 2-fluorine-18-fluoro-deoxy-glucose (2-[18F]FDG) positron emission tomography-computed tomography (PET/CT) is recommended for assessment of nodal and distant disease (Cibula et al. 2023). Pre-treatment 2-[18F]FDG-PET/CT has been shown useful not only for staging purposes, but also for providing prognostic information and to predict response to therapy in CSCC (Wang et al. 2021; Min et al. 2021).

Since both PET and MRI independently have a role in CSCC, 2-[18F]FDG-PET/MRI enabling simultaneous assessment of metabolism and functional imaging parameters with MRI may potentially have advantages in CSCC staging. PET/MRI has in single studies showed a higher detection rate of vaginal invasion, uterine invasion, bladder invasion, and cervical invasion than PET/CT, stand-alone MRI, and stand-alone CT (Zhu et al. 2021). In addition, the same study showed significantly higher sensitivity (94.7%), specificity (93.3%), and accuracy (93.9%) of PET/MRI in the detection of lymph node metastasis compared to PET/CT, MRI and CT (Zhu et al. 2021). 2-[18F]FDG-PET/MRI may be of use also in early response assessment during treatment, with the advantage of simultaneous MRI to improve localization and characterization of disease (Vojtisek et al. 2021). Consequently, assessment of imaging parameters in 2-[18F]FDG-PET/MRI performed before and early during treatment has the potential to improve prediction of radiotherapy outcomes. If imaging before start of treatment or early in treatment predicts tumor response to therapy, treatment adaptations to improve outcome or reduce the risk of side effects could be feasible.

In the present study, we investigated semantic 2-[18F]FDG-PET/MRI imaging features and their association with outcome after curative radiotherapy in CSCC.

## Materials and methods

### The MORRIS trial

The MORRIS trial—Multimodal Monitoring of Radiotherapy Response in Squamous Cell Cancer (MORRIS; NCT02379039)—was a prospective single-center observational study open to recruitment between 2015 and 2022. 110 patients with SCC in head and neck, anus, and uterine cervix, intended for definitive radiotherapy were included. The specific aim was to predict outcome two years after radiotherapy based on imaging, histopathology and metabolomic biomarkers at baseline and one week into radiotherapy. For all patients independent of tumor site, the radiotherapy was prescribed according to the national care guidelines on curative treatment for that specific diagnosis and no treatment interventions were made in the trial.

For CSCC the curative radiotherapy included conventionally fractionated radiotherapy to pelvic fields to 46–50 Gy followed by a boost delivered with pulsed dose rate (PDR) brachytherapy of 2 fractions à 15 Gy (EQD2, equivalent dose obtained using a 2 Gy fraction dose, of a minimum of 89 Gy was desired). In cases where brachytherapy was judged not suitable, an external radiotherapy boost to the tumor volume of a total of 66–68 Gy was delivered. Concomitant weekly Cisplatin 40 mg/m^2^ during radiotherapy was prescribed to those fit for the chemotherapy.

### Study participants

The participants of the present study consisted of an initial cohort of twelve CSCC patients from the MORRIS trial (MORRIS; NCT02379039), approved by the institutional review board (EPN2015/117-31), with the key inclusion criteria: previously untreated CSCC verified by histopathology or cytology, tumor radiologically visible and accessible for biopsy, planned for radiotherapy with curative intent, and minimum 20 years of age. Key exclusion criteria were contraindications to MRI, Gadolinium contrast agents or 2-[18F]FDG-PET.

All eligible MORRIS participants with CSCC and completed imaging work-up were included, resulting in 11 participants in the present study. Patient characteristics are shown in Table 1.

**Table 1:** Patient characteristics

Age at diagnosis	Gravidity	Parity	Tobacco use	FIGO stage	Histopathological grade	HPV +	
81	2	2	0	MA	Low-grade		1
48	0	^0^	0	IB2	High-grade	n/a	
81	2	2	1	NIB	High-grade	1	
51	3	2	0	MA2	High-grade	n/a	
60	2	2	1	MIB	High-grade		1
82	3	3	1	MIB	High-grade		1
28	0	0	0	IB2	High-grade	n/a	
53	4	^4^	0	MB, IllClr	Intermediate-grade		1
36	5	3	1	IB2, MIC2r	Intermediate-grade	n/a	
42	5	2	1	IBl/MIClr	Low-grade		
35	3	1	0	IB2	High-grade	n/a	

0 = no, 1 = yes, n/a = data not available

### Imaging protocol

The imaging consisted of baseline 2-[18F]FDG-PET/MRI performed one week before start of radiotherapy and early treatment response 2-[18F]FDG-PET/MRI performed after approximately one week (range 7–9 days) of RT. PET/MRI scanning was performed between 2016 and 2021 with an integrated hybrid PET/MRI 3.0 T scanner. The imaging protocols for 2-[18F]FDG-PET and pelvic MRI were designed according to current clinical routine protocols. The 2-[18F]FDG-PET was performed after 6 h of fasting and 1 h after intravenous administration of 2-[18F]FDG 4 MBq/kg body weight. MRI included T1-weighted (T1W), dynamic contrast-enhanced T1W, and T2-weighted (T2W) sequences, and Echo Planar diffusion-weighted imaging (EPI-DWI) with spatially selective excitation and *b* values 9, 200, 800 s/mm^2^. All baseline and early treatment response PET/MRI examinations were performed the day before tumor biopsies to avoid any false positive reactive uptakes.

### Imaging evaluation

A double licensed radiologist and nuclear medicine physician with > 10 years’ experience (SS) together with a radiation oncologist with > 10 years’ experience (KS) made a manual consensus delineation/annotation of gross tumor volume (GTV) of the primary tumor. The delineation was based on information from T2W sequences and the ADC maps, sequentially collected at baseline and at early treatment response PET/MRI. Quantitative semantic imaging parameters for both baseline and early treatment response PET/MRI were then extracted by two radiation physicists (JJ, MZ), from the manual segmentations: GTV from T2W and ADC, perpendicular lesion diameters (ccxllxap) measured in T2W and ADC, SUV_max_, mean SUV (SUV_mean_) and FTV measured from MRI attenuation-corrected PET data (static MRAC 30 min). Additional delta (change) in parameters between early response and baseline were calculated. For comparison, morphological measurements of tumors at baseline and early response assessment according to RECIST 1.1 (Eisenhauer et al. 2009) were also performed by SS. All imaging evaluations were performed independently and prospectively without knowledge of clinical outcome.

### Follow-up and statistics

All study participants had at least 24 months clinical follow-up. The evaluated treatment outcome was progression during follow-up and is represented by the categorical dichotomous parameter local relapse (yes/no). The primary endpoint for the trial was assessed by 2-[18F]FDG-PET/MRI and clinical examination after 3 months to assess early outcome. From six months to two years the outcome was evaluated clinically every 3rd month.

For patients without post radiotherapy remission, the time was set to 0. If the patient had no sign of relapse at end of follow up, the patient was censored at that time point. Descriptive statistics were used due to the small sample size. However, an exploratory logistic regression analysis of baseline SUV_max_, SUV_mean_, FTV, ADC, and delta SUV_max_, delta SUV_mean_, delta FTV and delta ADC as predictors for dichotomous outcome relapse was performed.

## Results

**Fig. 1:**
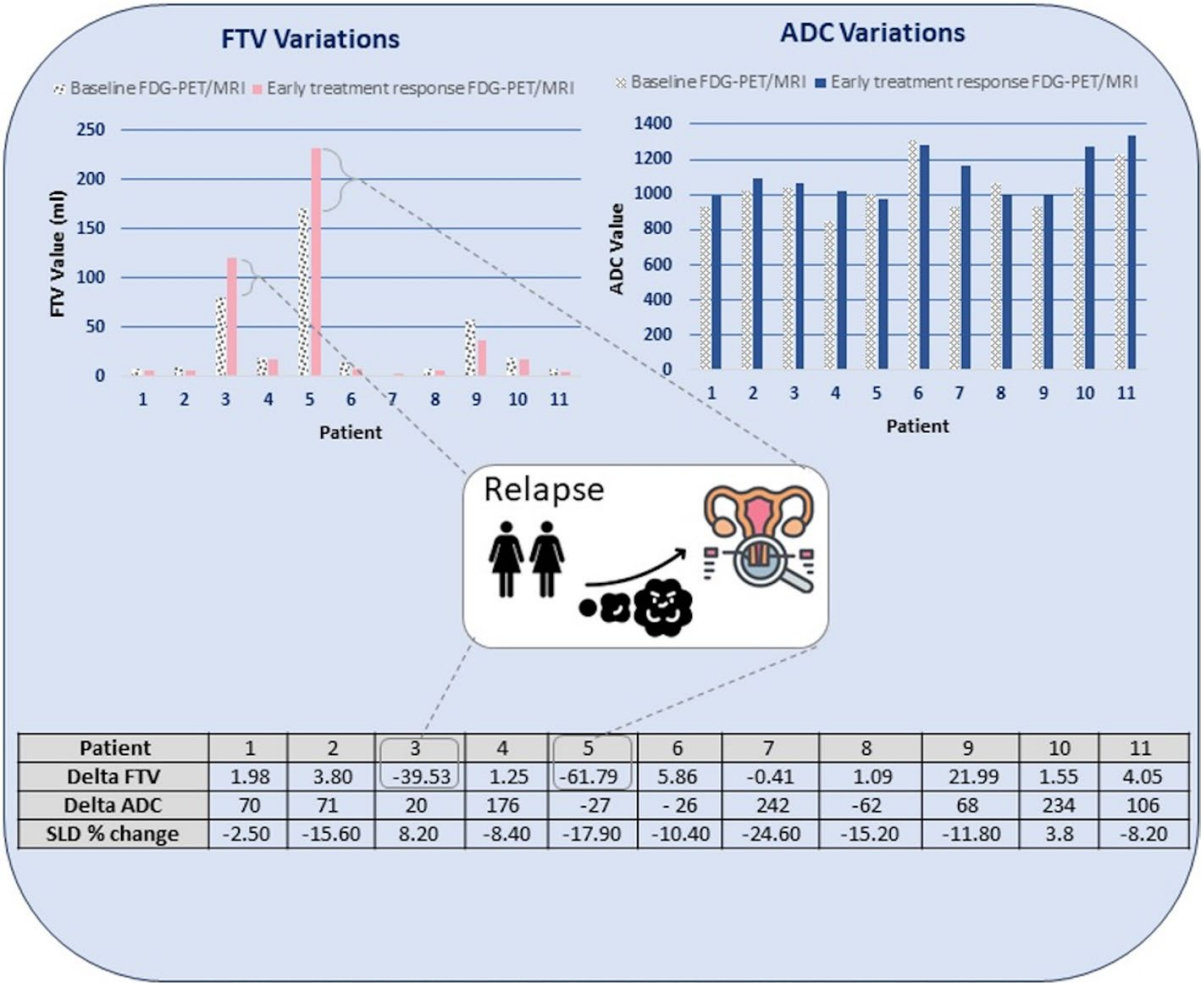
In the two participants with recurrent disease, there was a tendency toward higher FTV at baseline and at 1 week, as well as negative delta FTV (higher FTV after 1 week of radiotherapy)

During the follow-up period of minimum 24 months, relapse occurred in 2/11 study participants, with higher FTV as the most conspicuous imaging characteristic compared to the group with no relapse, see Fig. 1.

Median baseline FTV for relapses was 126 cm^3^, while for no relapses 9.3 cm^3^. Median delta FTV in the relapse group was 50.7 cm^3^, representing an actual increase in metabolically active volume, while median delta FTV in the no relapse group was − 2.0 cm^3^.

No difference in baseline ADC could be demonstrated between the groups; median baseline ADC for relapses was 1022 mm^2^/s, while for no relapses 1021 mm^2^/s. However, median delta ADC in the relapse group was − 3.5 mm^2^/s, representing a slight increase in diffusion restriction, while median delta ADC in the no relapse group was 71 mm^2^/s.

**Fig. 2:**
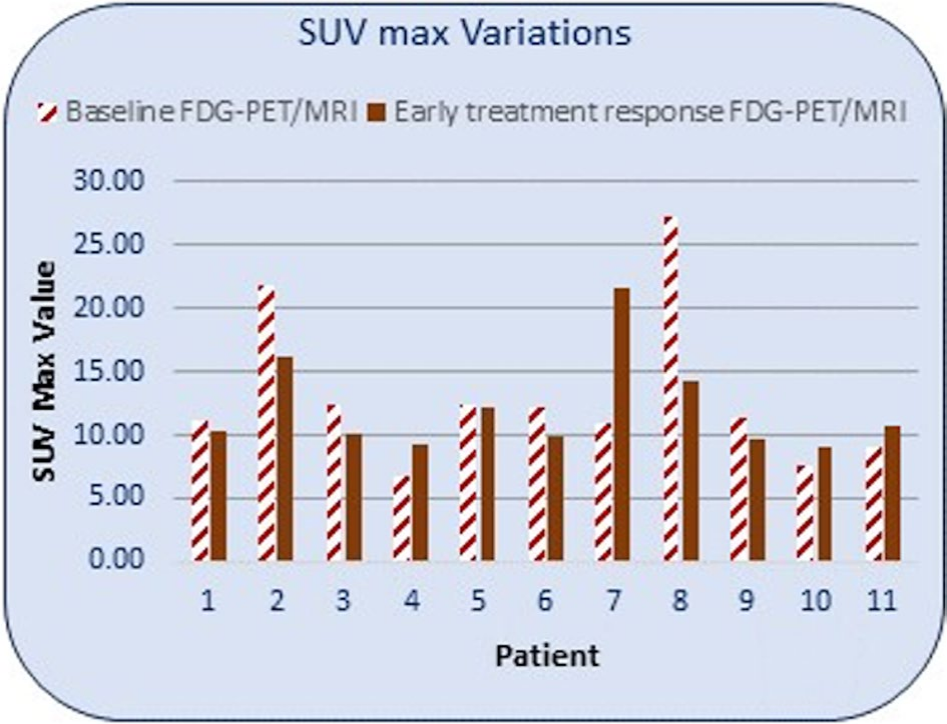
SUV_max_ variations

No difference in baseline SUV_max_ could be found between the groups; median baseline SUV_max_ was 12.4 g/ml for the relapse group, compared to 11.2 g/ml for the no relapse group, see Fig. 2.

None of the other imaging parameters showed any visual tendency towards a difference between the groups.

Exploratory logistic regression analysis was performed, with baseline SUV_max_, SUV_mean_, FTV, ADC, and delta SUV_max_, delta SUV_mean_ delta FTV and delta ADC as predictors for dichotomous outcome relapse. Baseline FTV (*p* = 0.004) and delta FTV (*p* = 0.002) turned out to be significantly higher in the relapse group. No significant difference was found for delta ADC (*p* = 0.095).

For comparison with standard predictive parameters, median clinical FIGO stage for relapses was stage III, while for the no relapse group stage I.

The baseline and early response imaging characteristics of the tumors are shown in Table 2. Quantitative parameters are demonstrated in descriptive statistics median, interquartile range (IQR), minimum and maximum, due to the limited number of cases, where the data may not be normally distributed.

**Table 2:** Lesion-based quantitative PET/MRI characteristics at baseline and early response evaluation, and additional delta parameters

Description	Median (IQR)	Minimum	Maximum
*Baseline*			
SUV_max_	11.33 (10.09 to 12–49)	6.72	27.31
SUV_mean_	7.39 (6.56–8.31)	4.56	18.76
FTV	14.36 (8.15–38.60)	1.98	171.11
T2_LL	61.01 (51.56–67.03)	31.36	82.50
T2_AP	39.10 (38.24–56.29)	29.12	76.48
T2 CC	30 (28.5–40.5)	27	87
ADC LL	45.70 (40.42–55.54)	13.35	94.21
ADC AP	40.07 (31.99–57.30)	10.54	77.34
ADC CC	30 (25–42.5)	10	75
ADC	1021 (929.5–1053)	848	1312
*Early response evaluation*			
SUV_max_	10.32 (9.84–13.17)	8.97	21.63
SUV_mean_	6.97 (6.16–8.41)	5.71	14.27
FTV	8.49 (5.6–26.9)	2.39	232.91
T2_LL	51.56 (44.25–62.52)	28.79	79.49
T2_AP	38.67 (32.44–47.91)	19.77	72.62
T2 CC	39 (31.5–39)	15	78
ADC LL	40.07 (34.45–62.34)	18.98	83.67
ADC AP	35.85 (29.88–49.21)	22.50	73.83
ADC CC	30 (22–40)	15	65.02
ADC	1062 (1001–1221)	975	1333
*Delta*			
SUV_max_	0.91 (− 1.52 to 2.32)	− 10.59	13.12
SUV_mean_	0.95 (− 0.90 to 1.26)	− 6.88	9.37
FTV	1.54 (0.33–3.9)	− 61.79	21.99
T2_LL	3.86 (2.79–7.94)	− 2.15	16.22
T2_AP	3.86 (− 0.21 to 7.94)	− 5.59	22.34
T2 CC	(− 6.00 to 7.5)	− 12	12
ADC LL	2.10 (− 5.97 to 7.38)	− 15.71	20.39
ADC AP	0.00 (− 5.62 to 6.67)	− 11.95	20.39
ADC CC	5.00 (0.00–10)	− 15	25
ADC	70 (41–141)	− 27	242

SUV max = maximum standardized uptake value, SUV mean = mean standardized uptake value, FTV = functional tumor volume, T2 LL = maximum lateral-lateral tumor diameter measured on T2W, T2 AP = maximum anteroposterior tumor diameter measured on T2W, T2 CC = craniocaudal tumor diameter measured on T2W, ADC LL = laterolateral tumor diameter measured on apparent diffusion coefficient map, ADC AP = anteroposterior tumor diameter measured on apparent diffusion coefficient map, ADC CC = craniocaudal tumor diameter measured on apparent diffusion coefficient map, ADC = tumor apparent diffusion coefficient value

**Fig. 3:**
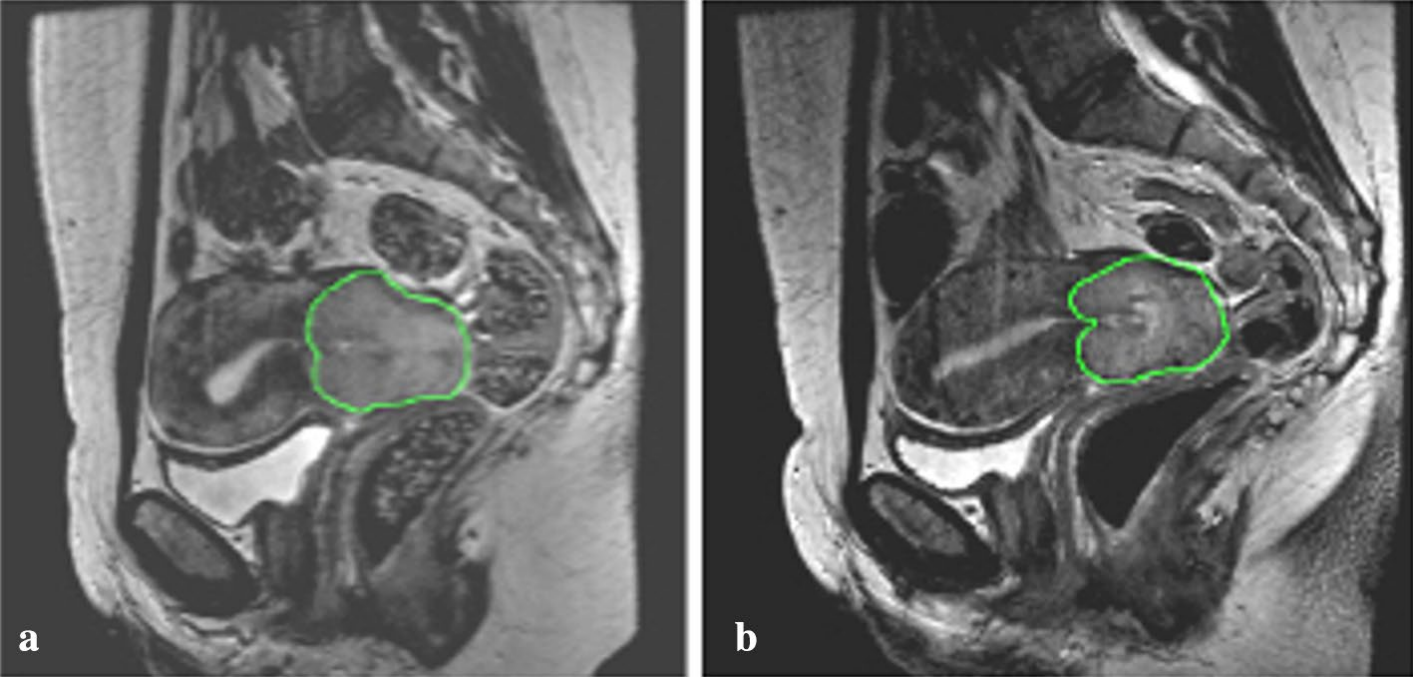
**a** Baseline GTV delineation on T2W MRI for one of the study participants. **b** Early treatment response evaluation GTV on T2W MRI, single modality, for the same study participant, showing a minor volume reduction on visual assessment

All study participants were assessed according to RECIST 1.1. Visual measurable decrease in tumor size was found in 9/11 participants (range − 2.5 to − 24.6% change in sum of largest diameters (SLD), %SLD change), while 2/11 showed an increase in %SLD change (8.2% and 3.8%, respectively). Decrease in %SLD change was less pronounced, median − 4.9%, in the relapse group compared to the no relapse group, which had a median reduction of − 10.4%, see Fig. 3.

All participants had target lesions (primary tumor) > 10 mm, but none qualified as significantly reduced or increased after one week of radiotherapy, hence all participants were at this stage classified as non-responders/stable disease according to RECIST 1.1. However, it should be noted that the RECIST criteria were not originally proposed for local tumor assessment.

## Discussion

Our results indicate that for CSCC treated with radiotherapy, early treatment response assessment with 2-[18F]FDG-PET/MRI of both functional and morphological tumor properties show changes in functional imaging parameters that may possibly predict clinical outcome. In this pilot cohort we found that baseline FTV, delta FTV and delta ADC may be potentially predictive parameters. Several studies support the assumption that early response imaging may predict tumor response to therapy. In a systematic review and meta-analysis from 2021, diffusion-weighted imaging (DWI) and particularly delta-apparent diffusion coefficient (ADC) emerged as suitable biomarkers of early treatment response in cervical carcinoma, where the pooled mean delta-ADC percentage was significantly higher in responders than in non-responders (Harry et al. 2021), which is in line with our results. Other studies have shown that various imaging parameters from baseline MRI and 2-[18F]FDG-PET/computed tomography (CT), in particular baseline tumor volume, may contribute to prediction of response to concurrent chemoradiotherapy in cervical cancer (Min et al. 2021). A study on mid-treatment 2-[18F]FDG-PET/MRI after five weeks of concurrent chemoradiotherapy in cervical cancer showed a statistically significant difference between responders and non-responders in mid-functional tumor volume (FTV), mid-total lesion glycolysis (TLG), mid-tumor size, and % change in maximum standardized uptake value (SUV_max_) (Vojtisek et al. 2021). A study on locally advanced cervical cancer patients receiving concurrent chemoradiotherapy or radiotherapy showed that baseline primary tumor TLG, derived from FTV, and lymph node SUV_max_ may be important prognostic biomarkers for survival (Wang et al. 2021). Furthermore, similar studies on other SCCs have demonstrated that baseline 2-[18F]FDG-PET may predict prognosis, for instance in esophageal cancer, where a baseline 2-[18F]FDG-PET SUV_max_ cut-off > 12.7  g/mL independently predicted early relapse after surgery with curative intent (Mantziari et al. 2020). In our study, we could not show any association between SUV_max_ and outcome prediction; however, FTV appeared to be linked to relapse, as could be expected from previous data. Interestingly, delta FTV showed an increase in metabolically active tumor volume after one week of radiotherapy, implying a possible reactive uptake from inflammation caused by radiotherapy, or an activation of tumor growth. This will be investigated in future histopathological analyzes of the tumor biopsies performed immediately after each 2-[18F]FDG-PET/MRI examination according to the study protocol.

In summary, our current results, which must be interpreted with caution due to the size of the study population, are in line with previous data where FTV and FTV-derived parameter TLG (Wang et al. 2021; Vojtisek et al. 2021), as well as ADC seem to be of particular predictive importance (Min et al. 2021; Harry et al. 2021).

Extended imaging information may be obtained not only from semantic parameters as described above, but also from radiomic features. A recent study on 19 patients with CSCC examined with 2-[18F]FDG-PET/CT and -PET/MRI radiomics showed significant difference between features in local and metastatic tumors including FTV, TLG, and entropy on PET from PET/CT; FTV and TLG on PET from PET/MRI; compactness and entropy on T2W; and entropy on ADC images (Esfahani et al. 2022). This further supports the results from our study, emphasizing the predictive effect of the metabolically active tumor volume. It should be stressed that the metabolically active tumor volume is not necessarily equivalent to the structural volume, which could be affected by tumor necrosis, and therefore the structural volume is not as reliable as imaging biomarker.

In another recent retrospective study on 2-[18F]FDG PET/CT radiomics with 50 patients, SUV_peak_ and the textural analysis feature gray-level run-length matrix, were found to predict overall survival in locally advanced CSCC (Alencar et al. 2022).

We believe that imaging analysis may be further improved with the radiomics approach but for this to be meaningful, large datasets are required. The observational cohort design of our study included the intention of performing radiomics analysis; however, at present due to the small number of included patients, radiomics analysis is not feasible on our data.

At early assessment, none of the patients in our study presented a change in tumor size consistent with partial response according to RECIST 1.1. Our results support the statement that RECIST 1.1 is not suitable for early treatment response evaluation of radiotherapy in CSCC.

Beyond RECIST 1.1, visual decrease in tumor size was present in the majority (9/11) of participants. The two cases with slightly increased tumor diameter could possibly be explained by measurement errors or methodological limitations such as differences in angulation, automated scanner-generated ADC maps and other post-processing procedures. However, there may be other causes in which there would be a true increase in tumor diameter, such as immuno-related responses to RT—inflammation or regeneration of cancer cells due to changes in the tumor micro-environment (He et al. 2023).

This study has several limitations, above all the small sample size which may only detect potential trends in the results, intended for further investigations in future more large-scale studies. This said, the amount of imaging data and correlating histopathological data, as well as access to clinical follow-up, makes the multidisciplinary study concept otherwise well designed. In addition, ample variation in the data between the different patients supports the hypothesis that 2-[18F]FDG-PET/MRI can be used to individualize treatment in CSCC.

For practical reasons, the clinical implementation of imaging biomarkers and radiomics analysis for outcome prediction and prognosis in CSCC with advanced imaging modalities such as PET/CT and PET/MRI, lies far ahead. Except for the extensive scientific investigations and validation of results that are warranted, the availability of PET/CT and even more so PET/MRI, is limited today. Should our preliminary results be confirmed in larger prospective studies, the benefit of using imaging biomarkers for direction of individualized treatment and improved survival rates would likely outweigh the costs of the PET/CT and PET/MRI investments, compared to the costs of suboptimal treatment regimens with unwanted adverse effects, less efficient cancer therapy, and the associated increased suffering, morbidity, and mortality.

## Conclusion

In conclusion, early response imaging with 2-[18F]FDG-PET/MRI may have the potential to predict radiotherapy outcome in CSCC but this needs to be confirmed in larger studies. Validation of our observations and correlation with survival data is warranted.

From our data, particularly delta FTV and delta ADC should be further investigated.

RECIST 1.1 does not seem suitable for early treatment response evaluation in CSCC after one week of radiotherapy.

## Data Availability

All data generated or analyzed during this study are included in this published article.

## Abbreviations

FDG: Fluoro-deoxy-glucose
PET: Positron emission tomography
MRI: Magnetic resonance imaging
CSCC: Cervical squamous cell carcinoma
FTV: Functional tumor volume
ADC: Apparent diffusion coefficient
DWI: Diffusion-weighted imaging
CT: Computed tomography
TLG: Total lesion glycolysis
SUV_max_: Maximum standardized uptake value
T1W: T1-weighted
T2W: T2-weighted
EPI-DWI: Echo planar DWI
HPV: Human papilloma virus
GTV: Gross tumor volume
SUV_mean_: Mean standardized uptake value
IQR: Interquartile range
SLD: Sum of largest diameters
LL: Lateral-lateral
AP: Anterioposterior
CC: Craniocaudal
